# Supplementary material for: Clustering induces switching between phoretic and osmotic propulsion in active colloidal rafts
Source: Nat Commun. 2024 Jul 6;15:5666. doi: 10.1038/s41467-024-49977-5 (PMC11227538; doi:10.1038/s41467-024-49977-5)
Supplement: Supplementary file 1 — Supplementary file [file 41467_2024_49977_MOESM1_ESM.pdf]

Supplementary Information
Clustering induces switching between phoretic and osmotic propulsion in active colloidal rafts

Dolachai Boniface,[1] Sergi G. Leyva,[1,2] Ignacio Pagonabarraga,[1,2] and Pietro Tierno[1,2]
[1]*Departament de Física de la Matèria Condensada, Universitat de Barcelona, 08028, Spain*
[2]*University of Barcelona Institute of Complex Systems (UBICS), 08028, Barcelona, Spain*

SUPPLEMENTARY FIGURES

Supplementary Figure 1

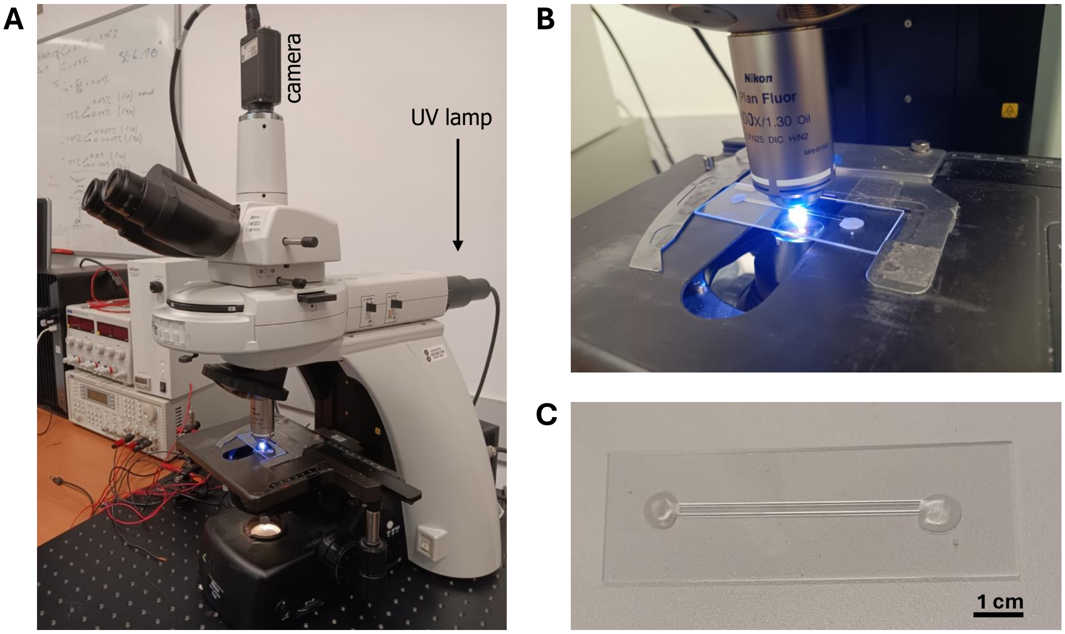

Supplementary Fig. 1. (**A**) Image of the experimental setup showing the upright optical microscope with a with a charge-coupled device camera and an epifluorescent tower. The light is provided with a commercial mercury fiber illumination system passed through a band-pass filter. (**B**) Detail of the microscope sample stage with the experimental cell containing the colloidal suspension and illuminated by blue light (wavelength $\lambda = 450 - 490$ nm) passing through a Nikon $100\times$ microscope objective. (**C**) Image showing the experimental cell made of a commercial microscope slide with a rectangular glass microtube (inner dimensions 2×0.1 mm) which is sealed on both sides by wax at the atmospheric pressure.

Supplementary Figure 2

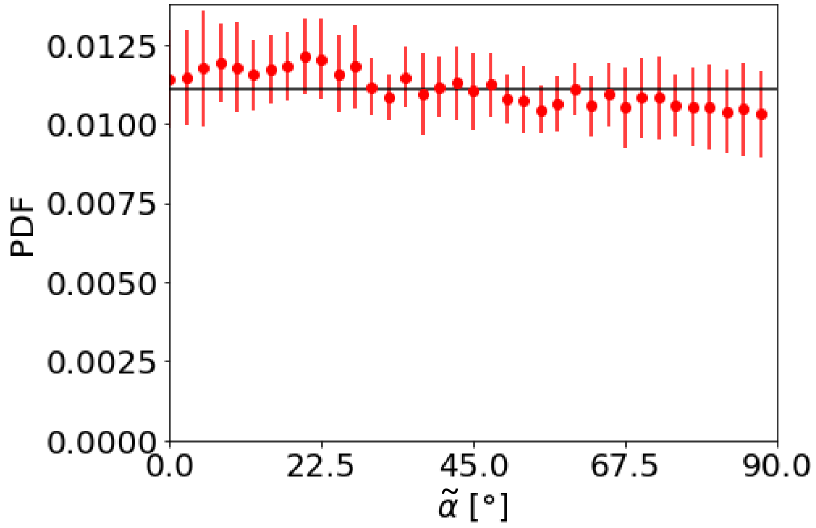

Supplementary Fig. 2. Probability distribution function of the angle $\tilde{\alpha}$, formed between the short axis and the velocity vector of a colloidal raft. The error bars in the data results from the statistical average of different set of data.

Supplementary Figure 3

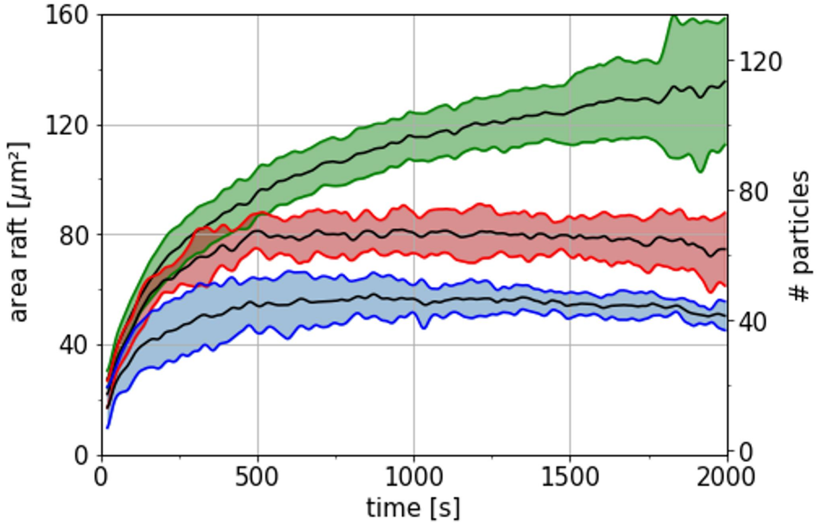

Supplementary Fig. 3. Evolution over time of the raft size for two light intensities and above two different substrates. The green curve corresponds to a light intensity of $I = 125\mathrm{mW\,cm^{-2}}$ and a glass substrate, the red one to a light intensity of $I = 60\mathrm{mW\,cm^{-2}}$ and a glass substrate, and the blue curve to a light intensity of $I = 125\mathrm{mW\,cm^{-2}}$ and a polystyrene substrate. For all the experimental data the black lines are corresponding averages and the shaded region denotes the confidence interval for $P = 0.95$. The associated errors depicted as shaded region results from the statistical average of different set of data.

Supplementary Figure 4

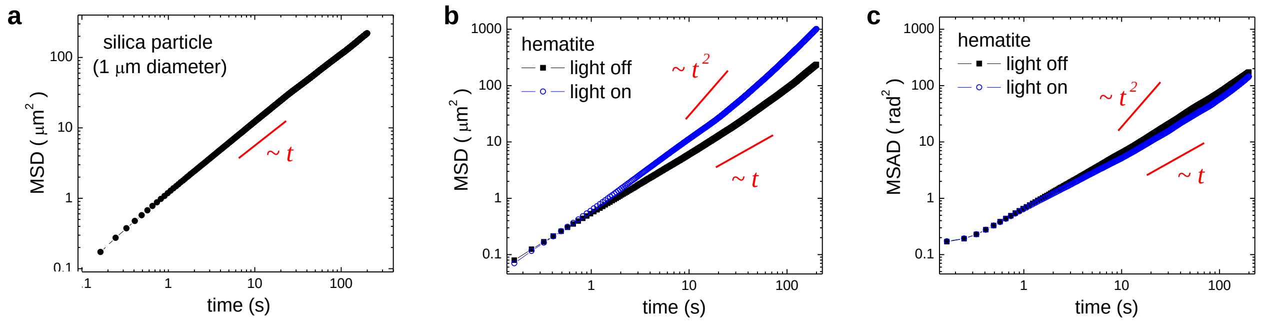

Supplementary Fig. 4. (a) Translation mean square displacement of a passive silica sphere with 1μm diameter and (b) of a hematite particle in the absence of light (black squares) and with blue light (blue circles). (c) Angular mean square displacement of the hematite particle in the absence (black squares) and presence (blue circles) of light. In each graph the slopes that indicate diffusive ($\sim t$) and ballistic ($\sim t^2$) regimes are shown in red.

Supplementary Figure 5

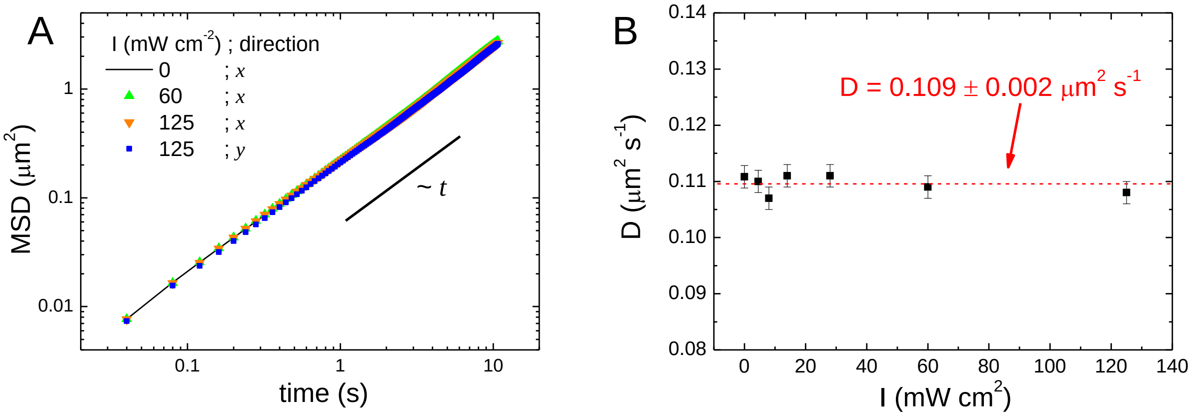

Supplementary Fig. 5. (A) One dimensional translational mean square displacements (MSDs) as a function of time of passive silica particles with 2μm diameter and subjected to blue light at different light intensities I. For $I = 125\text{mW cm}^{-2}$ the MSDs are shown along the two orthogonal directions (x, y), which demonstrate the isotropic diffusion and the absence of net drift. All the curves display standard diffusive dynamics and overlap, indicating the absence of any temperature variation. (B) Corresponding diffusion coefficient (D) as a function of the light intensity (I) extracted from the MSDs of 10 different particles and averaged along the two orthogonal direction (x, y) at each light intensity. The experimental data display small fluctuations within the error bars around a mean value of $D = 0.109 \pm 0.002\text{mW s}^{-1}$. The error bars has been obtained from the statistical average of different measurments.

Supplementary Figure 6

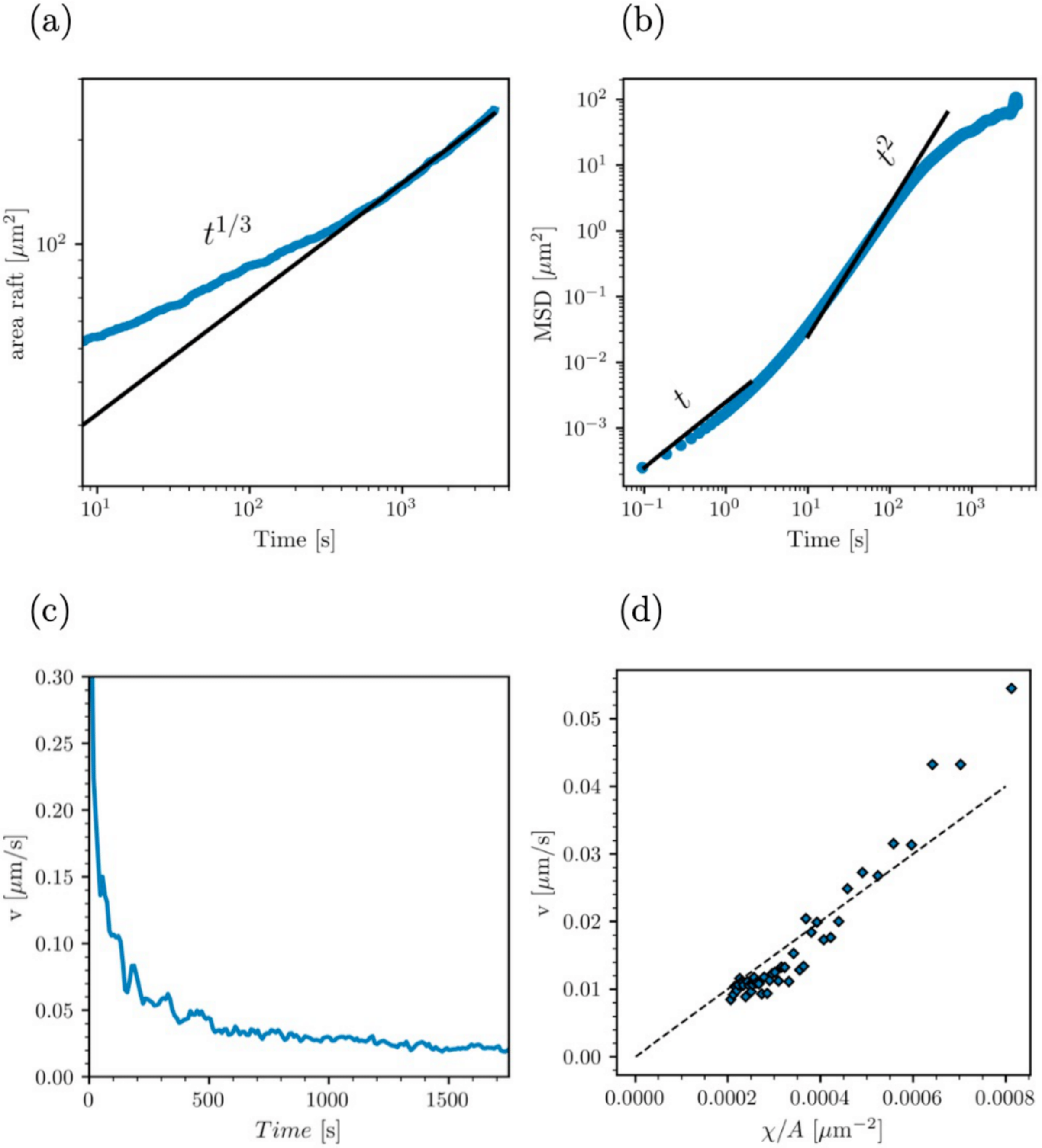

Supplementary Fig. 6. (a-d) Simulation results illustrating: (a) The evolution of the raft area versus time, which follows the power law $t^{1/3}$ at long time. (b) Mean square displacement, which corresponds to the typical self-propulsion behavior: diffusion at small times and ballistic motion at large times. (c) Time evolution of the raft velocity and (d) raft velocity as a function of the parameter χ/A, which exhibits a linear dependence.

Supplementary Note 1: Diffusion of passive and actives particles.

In Supplementary Fig. 4(a-c) we show the measured translation mean square displacement (MSD) for the silica particle (a) and the translational and angular MSD (b) [MSAD, (c)] for the hematite particle without and with light. We compute both quantities using the expressions: MSD$\equiv\ \langle \Delta \boldsymbol{r}^2\rangle(\tau) = \frac{1}{N}\sum_{n=1}^{N}\langle(\boldsymbol{r}(t) - \boldsymbol{r}(t+\tau))^2\rangle_t$ and MSAD$\equiv\ \langle \Delta \boldsymbol{\theta}^2\rangle(\tau) = \frac{1}{N}\sum_{n=1}^{N}\langle(\boldsymbol{\theta}(t) - \boldsymbol{\theta}(t+\tau))^2\rangle_t$. Here $\langle ...\rangle_t$ is a time average, $\boldsymbol{r}(t)$ is the two-dimensional position of the particle, θ represents its orientation (for the hematite) and τ denotes the lag time.

Translation diffusion D. From the MSD of a single particle, we determine the translation diffusion coefficient D in the long time limit by using the formula MSD$(\tau) \simeq 4D\tau$. For the 1 μm silica particles, and the hematite particle without blue light, this estimation is straightforward, since the transport is purely diffusive, $D_p = 0.29 \pm 0.07\,\mu\mathrm{m}^2\,\mathrm{s}^{-1}$, D_a(No light) $= 0.16 \pm 0.04\,\mu\mathrm{m}^2\,\mathrm{s}^{-1}$.

However, when the hematite particle is exposed to blue light, this transport becomes superdiffusive. The slope of the MSD is slightly above one (slope ~ 1.3). If we try to fit a linear behavior, we find a qualitative diffusion coefficient $D_a(\mathrm{light}) = 0.26 \pm 0.07\,\mu\mathrm{m}^2\,\mathrm{s}^{-1}$, about two times larger than without blue light. This effect is probably due to the phoretic flow. A slight asymmetry or inhomogeneity of the hematite shape or surface can induce this self-propulsion. Indeed in a previous work [1] it was found that after an etching treatment with acid, 60% of the hematite particles were able to self-propel.

Angular diffusion coefficient D_a. We also measure the MSAD for the hematite particle both in the absence and in the presence of light. The prolate shape of the hematite particles allows to determine their instantaneous orientation, and by extension an angular diffusion. Similarly to the translation diffusion, we distinguish the case with and without blue light

$$D_a(\mathrm{No\ light}) = 0.33 \pm 0.08\ \mathrm{rad}^2.\mathrm{s}^{-1}, \qquad D_a(\mathrm{light}) = 0.29 \pm 0.07\ \mathrm{rad}^2.\mathrm{s}^{-1}.$$

Exposing the hematite to light does not induce a superdiffusive behavior thus, we find a similar value for both cases.

Supplementary Note 2: Description of the measurement of different observables.

- **Raft velocity** v_c: For the cluster, we use particle tracking routines [2] to obtain the positions (x, y) of the central hematite particle over time. To determine the velocity of the cluster, we apply Gaussian smoothing to the detected cluster area using a standard deviation $\sigma = 3$ s. The choice of this standard deviation is justified by the MSD analysis, since it corresponds to the onset of the ballistic regime, while the diffusive regime occurs at time scales Δt below 1 second. To derive the smoothed x and y positions, we interpolate them using a third degree B-spline function. The first derivative gives v_x and v_y, and their norm give the magnitude of raft velocity $v_c = \sqrt{v_x^2 + v_y^2}$.

- **Relative velocity Δv_r for the hematite-silica pair interaction**: We use particle tracking to obtain the positions of both the hematite and the silica particles, from which we extract the relative distance Δr. Similar to the previous treatment, Δr undergoes a Gaussian smoothing with a short standard deviation $\sigma = 0.01$ s, and the relative velocity Δv_r is obtained through the derivative of a third degree B-spline interpolating function.

- **Surface area** A: The first challenge in determining the size of a cluster lies in discerning which particles belong or not to the cluster, which can be difficult for a dense mixture of passive colloids. To address this issue, we take advantage of the different dynamics between the colloidal raft, which moves slowly, and the thermally free silica colloids, diffusing rapidly. The main idea is to consider the "average" image of the cluster over a time range long enough for the free particles to disappear, yet short enough to prevent significant size evolution for the cluster. In our case, this time range spans 4 seconds (approximately 48 frames) centered around a reference frame.

  More specifically, all frames within this time range are cropped so that they mostly display the cluster. Subsequently, each frame is rotated and shifted to achieve the best correlation with the reference cropped frame. The rotation angle is determined with the Fourier transform of the two frame to correlate [3]. Upon rotation to the appropriate angle, the optimal shift correlation is determined and applied. These adjustments aim to align the cluster's image closely with the reference frame, resulting in a clear depiction of it in the average image, while the free silica colloids disappear. The images presented in Figure 1.D were obtained using this technique.

  From the average image, we employ the *Canny* edge detection algorithm to determine the contour of the cluster, and the function *floodfill* to fill the contour. Both algorithms are from the library *OpenCV* . From the binary image of the cluster, we extract the surface area A and center of mass $O(x_o, y_o)$.

- **Radius a of the colloidal raft**: The 'radius' a of the cluster is then computed from the area using the formula $a = \sqrt{A/\pi}$.
- **Vector b and angle β**: With the central hematite position tracking (x,y), we compute $\mathbf{b} = (x - x_c, y - y_c)$ from the cluster's center of mass. Utilizing the previously determined values v_x and v_y, we derive the velocity vector $\boldsymbol{v}$, from which we calculate the angle $\beta = \widehat{(\boldsymbol{v}, \boldsymbol{b})}$.

SUPPLEMENTARY TABLE

Name	Symbol	Value used
Number of active particles (dumbbells)	N_a	1
Number of passive particles	N_p	700
Area fraction of particles	A_φ	0.06
Diameter of passive particle	σ_p	1μm
Diameter of active particle in dumbbell	σ_a	1.3μm
Spring constant of active dumbbell	k	100
Rest length of the spring constant	l_0	0.5μm
Péclet of active particles	Pe_a	60
Péclet of passive particles	Pe_p	53.8
Ratio between mobilities	$\bar{\mu}$	10
Rescaled characteristic velocity	$v_0\bar{\mu}$	11.6 μ m s^{-1}
Characteristic time scale	τ_c	0.086 s
Total simulation time	t_{tot}	5160 s
Dimensionless ratio	$\mu_a k\sigma_p/v_0\bar{\mu}$	50
Dimensionless ratio	$\epsilon_{a,p}\mu_{a,p}/v_0\bar{\mu}$	10

Supplementary Table. I. List of the numerical values of the different parameters used in the simulations.

SUPPLEMENTARY REFERENCES

[1] J. Palacci et al. Photoactivated Colloidal Dockers for Cargo Transportation. *J. Am. Chem. Soc.*, **135**, 15978-15981 (2013).

[2] Crocker, J. C. & Grier, D. G. Methods of Digital Video Microscopy for Colloidal Studies. *J. Coll. Inter. Sci.*, **179**, 298-310 (1996).

[3] Wilson, C. A. & Theriot, J. A. A correlation-based approach to calculate rotation and translation of moving cells *IEEE Transactions on Image Processing*, **15**, 1939-1951 (2006).
