# Supplementary material for: Clustering induces switching between phoretic and osmotic propulsion in active colloidal rafts
Source: Nat Commun. 2024 Jul 6;15:5666. doi: 10.1038/s41467-024-49977-5 (PMC11227538; doi:10.1038/s41467-024-49977-5)
Supplement: Supplementary file 3 — Description of Additional Supplementary Files [file 41467_2024_49977_MOESM3_ESM.pdf]

Description of Additional Supplementary Files

File Name: Supplementary Movie 1

Description: This videoclip illustrates the formation of a colloidal raft composed of a central hematite particle and a growing shell of passive spheres. During growth, the raft displays self-propulsion. The aggregation has been induced by blue light (wavelength λ = 450 - 490 nm) at an intensity I = 125 mW cm^{-2}. The video has been accelerated 30 times, and it corresponds to Figure 1(b) of the main manuscript.

File Name: Supplementary Movie 2

Description: This videoclip illustrates the dynamics of a hematite particle and silica microspheres (1 μm diameter) dispersed in a hydrogen peroxide water solution and in the absence of any illumination. Both particles display standard diffusive dynamics without being affected by any phoretic flow.

File Name: Supplementary Movie 3

Description: In this videoclip we show the steady state dynamics (after ~10 min of illumination) and the absence of attraction between a hematite particle and the silica microspheres (1 μm) when dispersed in pure water (without hydrogen peroxide) and subjected to the strongest illumination power I = 125 mW cm^{-2}. In this case as well, these particles display standard diffusive dynamics without being affected by any phoretic flow.

File Name: Supplementary Movie 4

Description: Videoclip made from numerical simulations illustrating the aggregation and propulsion of a composite raft. In the video, the passive particles are illustrated by the blue disks, while the active one is a dimer composed of two orange disks. The input parameters are the same as the experimental ones, as described in the Method Section. Lengths on the sides are in μm.

File Name: Supplementary Movie 5

Description: Videoclip made from numerical simulations illustrating the long persistence length of a composite raft moving with the active particle at the rear. In the video, the passive particles are illustrated by the blue disks, while the active one is a dimer composed of two orange disks. Considering the clustering phenomenon, the input parameters are like the experimental one, as described in this supporting information. For the cluster propulsion, we impose the cluster to move with the active particle at the rear, and the velocity follows the formula, $v_{cl} = k\,\chi/\mathrm{A}$, where k = 90 μm^{-1} s^{-1} as determined in the experiments. Lengths on the sides are in μm.
